# Supplementary material for: Chondroprotective Actions of Selective COX-2 Inhibitors In Vivo: A Systematic Review
Source: Int J Mol Sci. 2020 Sep 22;21(18):6962. doi: 10.3390/ijms21186962 (PMC7555215; doi:10.3390/ijms21186962)
Supplement: Supplementary file 1 [file ijms-21-06962-s001.zip › ijms-931722-supplymentary revised done/bias assesment.pdf]

Supplementary Data: Syrcle risk of bias tool for *in vivo* animal studies

Mastbergen et al. (1):

Was the allocation sequence adequately generated and applied?

1. Did the investigators describe a random component in the sequence generation process?

   No

Were the groups similar at baseline, or were they adjusted for the confounders in the analysis?

1. Was the distribution of relevant baseline characteristics balanced for the intervention and control groups?

   Unclear

2. If relevant, did the investigators adequately adjust for unequal distribution of some relevant baseline characteristics in the analysis?

   Unclear

3. Was the timing of disease induction adequate?

   Yes

Was the allocation to the different groups adequately concealed during?

Could the investigator allocating the animals to intervention or control group not foresee assignment due to one of the following or equivalent methods?

Unclear

Were the animals randomly housed during the experiment?

1. Did the authors randomly place the cages or animals within the animal room/facility?

   Unclear

2. Is it unlikely that the outcome or the outcome measurement was influenced by not randomly housing the animals?

No

5) Were the caregivers and/or investigators blinded from knowledge which intervention each animal received during the experiment?

Was blinding of caregivers and investigators ensured, and was it unlikely that their blinding could have been broken?

Unclear

6) Were animals selected at random for outcome assessment?

Did the investigators randomly pick an animal during outcome assessment, or did they use a random component in the sequence generation for outcome assessment?

Unclear

7) Was the outcome assessor blinded?

1. Was blinding of the outcome assessor ensured, and was it unlikely that blinding could have been broken?

   Yes

2. Was the outcome assessor not blinded, but do review authors judge that the outcome is not likely to be influenced by lack of blinding?

   No

8) Were incomplete outcome data adequately addressed? (*)

1. Were all animals included in the analysis?

   Yes

2. Were the reasons for missing outcome data unlikely to be related to true outcome? (e.g., technical failure)

   No

3. Are missing outcome data balanced in numbers across intervention groups, with similar reasons for missing data across groups?

   No missing data

4. Are missing outcome data imputed using appropriate methods?

   No missing data

9. **Are reports of the study free of selective outcome reporting? (*)**

1. Was the study protocol available and were all of the study's pre-specified primary and secondary outcomes reported in the current manuscript?

   Yes

2.. Was the study protocol not available, but was it clear that the published report included all expected outcomes (i.e. comparing methods and results section)?

   Not applicable

10. **Was the study apparently free of other problems that could result in high risk of bias?**

1. Was the study free of contamination (pooling drugs)?

   No, but risk of biasing of the drugs used is low.

2. Was the study free of inappropriate influence of funders

   Yes

3. Was the study free of unit of analysis errors?

   Yes

4. Were design-specific risks of bias absent?

   Yes

Huh et al. (2) :

Was the allocation sequence adequately generated and applied?

1. Did the investigators describe a random component in the sequence generation process?

   No

Were the groups similar at baseline, or were they adjusted for the confounders in the analysis?

1. Was the distribution of relevant baseline characteristics balanced for the intervention and control groups?

   Unclear

2. If relevant, did the investigators adequately adjust for unequal distribution of some relevant baseline characteristics in the analysis?

   Unclear

3. Was the timing of disease induction adequate?

   Yes

Was the allocation to the different groups adequately concealed during?

Could the investigator allocating the animals to intervention or control group not foresee assignment due to one of the following or equivalent methods?

Unclear

Were the animals randomly housed during the experiment?

1. Did the authors randomly place the cages or animals within the animal room/facility?

   Unclear

2. Is it unlikely that the outcome or the outcome measurement was influenced by not randomly housing the animals?

   No

Were the caregivers and/or investigators blinded from knowledge which intervention each animal received during the experiment?

Was blinding of caregivers and investigators ensured, and was it unlikely that their blinding could have been broken?

Unclear

Were animals selected at random for outcome assessment?

Did the investigators randomly pick an animal during outcome assessment, or did they use a random component in the sequence generation for outcome assessment?

Unclear

Was the outcome assessor blinded?

1. Was blinding of the outcome assessor ensured, and was it unlikely that blinding could have been broken?

   Yes

2. Was the outcome assessor not blinded, but do review authors judge that the outcome is not likely to be influenced by lack of blinding?

   No

Were incomplete outcome data adequately addressed? (*)

1. Were all animals included in the analysis?

   Unclear

2. Were the reasons for missing outcome data unlikely to be related to true outcome? (e.g., technical failure)

   Unclear

3. Are missing outcome data balanced in numbers across intervention groups, with similar reasons for missing data across groups?

   Unclear

4. Are missing outcome data imputed using appropriate methods?

   Unclear

Are reports of the study free of selective outcome reporting? (*)

1. Was the study protocol available and were all of the study's pre-specified primary and secondary outcomes reported in the current manuscript?

   Yes

2.. Was the study protocol not available, but was it clear that the published report included all expected outcomes (i.e. comparing methods and results section)?

   Not applicable

Was the study apparently free of other problems that could result in high risk of bias?

1. Was the study free of contamination (pooling drugs)?

   Unclear

2. Was the study free of inappropriate influence of funders

   Yes

3. Was the study free of unit of analysis errors?

   Yes

4. Were design-specific risks of bias absent?

   Yes

Jones et al. (3) :

Was the allocation sequence adequately generated and applied?

1. Did the investigators describe a random component in the sequence generation process?

   Yes

Were the groups similar at baseline, or were they adjusted for the confounders in the analysis?

1. Was the distribution of relevant baseline characteristics balanced for the intervention and control groups?

Unclear

2. <u>If relevant, did the investigators adequately adjust for unequal distribution of some relevant baseline characteristics in the analysis?</u>

   Unclear

3. <u>Was the timing of disease induction adequate?</u>

   Yes

Was the allocation to the different groups adequately concealed during?

<u>Could the investigator allocating the animals to intervention or control group not foresee assignment due to one of the following or equivalent methods?</u>

Unclear

Were the animals randomly housed during the experiment?

4. <u>Did the authors randomly place the cages or animals within the animal room/facility?</u>

   Unclear

5. <u>Is it unlikely that the outcome or the outcome measurement was influenced by not randomly housing the animals?</u>

   No

Were the caregivers and/or investigators blinded from knowledge which intervention each animal received during the experiment?

<u>Was blinding of caregivers and investigators ensured, and was it unlikely that their blinding could have been broken?</u>

Unclear

Were animals selected at random for outcome assessment?

Did the investigators randomly pick an animal during outcome assessment, or did they use a random component in the sequence generation for outcome assessment?

Unclear

Was the outcome assessor blinded?

1. Was blinding of the outcome assessor ensured, and was it unlikely that blinding could have been broken?

   Yes

2. Was the outcome assessor not blinded, but do review authors judge that the outcome is not likely to be influenced by lack of blinding?

   No

Were incomplete outcome data adequately addressed? (*)

1. Were all animals included in the analysis?

   Yes

2. Were the reasons for missing outcome data unlikely to be related to true outcome? (e.g., technical failure)

   No

3. Are missing outcome data balanced in numbers across intervention groups, with similar reasons for missing data across groups?

   No missing data

4. Are missing outcome data imputed using appropriate methods?

   No missing data

Are reports of the study free of selective outcome reporting? (*)

1. Was the study protocol available and were all of the study's pre-specified primary and secondary outcomes reported in the current manuscript?

   Yes

2.. Was the study protocol not available, but was it clear that the published report included all expected outcomes (i.e. comparing methods and results section)?

Not applicable

Was the study apparently free of other problems that could result in high risk of bias?

1. Was the study free of contamination (pooling drugs)?

   No, but risk of bias of the used drugs is unlikely

2. Was the study free of inappropriate influence of funders

   Yes

3. Was the study free of unit of analysis errors?

   Yes

4. Were design-specific risks of bias absent?

   Yes

Fukai et al. (4):

Was the allocation sequence adequately generated and applied?

1. Did the investigators describe a random component in the sequence generation process?

   No

Were the groups similar at baseline, or were they adjusted for the confounders in the analysis?

1. Was the distribution of relevant baseline characteristics balanced for the intervention and control groups?

Yes

2. If relevant, did the investigators adequately adjust for unequal distribution of some relevant baseline characteristics in the analysis?

   Not applicable

3. Was the timing of disease induction adequate?

   Yes

Was the allocation to the different groups adequately concealed during?

Could the investigator allocating the animals to intervention or control group not foresee assignment due to one of the following or equivalent methods?

Unclear

Were the animals randomly housed during the experiment?

1. Did the authors randomly place the cages or animals within the animal room/facility?

   Unclear

2. Is it unlikely that the outcome or the outcome measurement was influenced by not randomly housing the animals?

   No

Were the caregivers and/or investigators blinded from knowledge which intervention each animal received during the experiment?

Was blinding of caregivers and investigators ensured, and was it unlikely that their blinding could have been broken?

Unclear

Were animals selected at random for outcome assessment?

Did the investigators randomly pick an animal during outcome assessment, or did they use a random component in the sequence generation for outcome assessment?

Unclear

Was the outcome assessor blinded?

1. Was blinding of the outcome assessor ensured, and was it unlikely that blinding could have been broken?

   Yes

2. Was the outcome assessor not blinded, but do review authors judge that the outcome is not likely to be influenced by lack of blinding?

   No

Were incomplete outcome data adequately addressed? (*)

1. Were all animals included in the analysis?

   Unclear

2. Were the reasons for missing outcome data unlikely to be related to true outcome? (e.g., technical failure)

   Unclear

3. Are missing outcome data balanced in numbers across intervention groups, with similar reasons for missing data across groups?

   Unclear

3. Are missing outcome data imputed using appropriate methods?

   Unclear

Are reports of the study free of selective outcome reporting? (*)

1. Was the study protocol available and were all of the study's pre-specified primary and secondary outcomes reported in the current manuscript?

   Yes

2.. Was the study protocol not available, but was it clear that the published report included all expected outcomes (i.e. comparing methods and results section)?

Not applicable

Was the study apparently free of other problems that could result in high risk of bias?

1. Was the study free of contamination (pooling drugs)?

   Unclear

2. Was the study free of inappropriate influence of funders

   Yes

3. Was the study free of unit of analysis errors?

   Yes

4. Were design-specific risks of bias absent?

   Yes

Ou et al.(5):

Was the allocation sequence adequately generated and applied?

1. Did the investigators describe a random component in the sequence generation process?

   Yes

Were the groups similar at baseline, or were they adjusted for the confounders in the analysis?

2. Was the distribution of relevant baseline characteristics balanced for the intervention and control groups?

   Unclear

3. <u>If relevant, did the investigators adequately adjust for unequal distribution of some relevant baseline characteristics in the analysis?</u>

   Unclear

4. <u>Was the timing of disease induction adequate?</u>

   Yes

Was the allocation to the different groups adequately concealed during?

<u>Could the investigator allocating the animals to intervention or control group not foresee assignment due to one of the following or equivalent methods?</u>

Unclear

Were the animals randomly housed during the experiment?

1. <u>Did the authors randomly place the cages or animals within the animal room/facility?</u>

   Unclear

2. <u>Is it unlikely that the outcome or the outcome measurement was influenced by not randomly housing the animals?</u>

   No

5) Were the caregivers and/or investigators blinded from knowledge which intervention each animal received during the experiment?

<u>Was blinding of caregivers and investigators ensured, and was it unlikely that their blinding could have been broken?</u>

Unclear

6) Were animals selected at random for outcome assessment?

Did the investigators randomly pick an animal during outcome assessment, or did they use a random component in the sequence generation for outcome assessment?

Unclear

7) Was the outcome assessor blinded?

1. Was blinding of the outcome assessor ensured, and was it unlikely that blinding could have been broken?

   No

2. Was the outcome assessor not blinded, but do review authors judge that the outcome is not likely to be influenced by lack of blinding?

   No, outcome may be influenced due to lack of blinding

8) Were incomplete outcome data adequately addressed? (*)

1. Were all animals included in the analysis?

   Unclear

2. Were the reasons for missing outcome data unlikely to be related to true outcome? (e.g., technical failure)

   Unclear

3. Are missing outcome data balanced in numbers across intervention groups, with similar reasons for missing data across groups?

   Unclear

3. Are missing outcome data imputed using appropriate methods?

   Unclear

9. **Are reports of the study free of selective outcome reporting? (*)**

1. Was the study protocol available and were all of the study’s pre-specified primary and secondary outcomes reported in the current manuscript?

   Yes

2.. Was the study protocol not available, but was it clear that the published report included all expected outcomes (i.e. comparing methods and results section)?

Not applicable

11. **Was the study apparently free of other problems that could result in high risk of bias?**

1. Was the study free of contamination (pooling drugs)?

   Unclear

2. Was the study free of inappropriate influence of funders

   Yes

3. Was the study free of unit of analysis errors?

   Yes

4. Were design-specific risks of bias absent?

   Yes

Ashkavand et al. (6):

Was the allocation sequence adequately generated and applied?

1. Did the investigators describe a random component in the sequence generation process?

   No

Were the groups similar at baseline, or were they adjusted for the confounders in the analysis?

1. Was the distribution of relevant baseline characteristics balanced for the intervention and control groups?

   Unclear

2. If relevant, did the investigators adequately adjust for unequal distribution of some relevant baseline characteristics in the analysis?

   Unclear

3. Was the timing of disease induction adequate?

   Yes

Was the allocation to the different groups adequately concealed during?

Could the investigator allocating the animals to intervention or control group not foresee assignment due to one of the following or equivalent methods?

Unclear

Were the animals randomly housed during the experiment?

1. Did the authors randomly place the cages or animals within the animal room/facility?

   Unclear

2. Is it unlikely that the outcome or the outcome measurement was influenced by not randomly housing the animals?

   No

5) Were the caregivers and/or investigators blinded from knowledge which intervention each animal received during the experiment?

Was blinding of caregivers and investigators ensured, and was it unlikely that their blinding could have been broken?

Unclear

6) Were animals selected at random for outcome assessment?

Did the investigators randomly pick an animal during outcome assessment, or did they use a random component in the sequence generation for outcome assessment?

Unclear

7) Was the outcome assessor blinded?

1. Was blinding of the outcome assessor ensured, and was it unlikely that blinding could have been broken?

   Unclear

2. Was the outcome assessor not blinded, but do review authors judge that the outcome is not likely to be influenced by lack of blinding?

   Unclear

8) Were incomplete outcome data adequately addressed? (*)

1. Were all animals included in the analysis?

   Yes

2. Were the reasons for missing outcome data unlikely to be related to true outcome? (e.g., technical failure)

   No

3. Are missing outcome data balanced in numbers across intervention groups, with similar reasons for missing data across groups?

No missing data

3. Are missing outcome data imputed using appropriate methods?

No missing data

9. **Are reports of the study free of selective outcome reporting? (*)**

1. Was the study protocol available and were all of the study's pre-specified primary and secondary outcomes reported in the current manuscript?

Yes

2.. Was the study protocol not available, but was it clear that the published report included all expected outcomes (i.e. comparing methods and results section)?

Not applicable

3. Was the study apparently free of other problems that could result in high risk of bias?

1. Was the study free of contamination (pooling drugs)?

Yes

2. Was the study free of inappropriate influence of funders

Yes

3. Was the study free of unit of analysis errors?

Yes

4. Were design-specific risks of bias absent?

Yes

Moon et al. (7)

Was the allocation sequence adequately generated and applied?

1. Did the investigators describe a random component in the sequence generation process?

   Yes

Were the groups similar at baseline, or were they adjusted for the confounders in the analysis?

2. Was the distribution of relevant baseline characteristics balanced for the intervention and control groups?

   Unclear

3. If relevant, did the investigators adequately adjust for unequal distribution of some relevant baseline characteristics in the analysis?

   Unclear

4. Was the timing of disease induction adequate?

   Yes

Was the allocation to the different groups adequately concealed during?

Could the investigator allocating the animals to intervention or control group not foresee assignment due to one of the following or equivalent methods?

Unclear

Were the animals randomly housed during the experiment?

1. Did the authors randomly place the cages or animals within the animal room/facility?

   Unclear

2. Is it unlikely that the outcome or the outcome measurement was influenced by not randomly housing the animals?

   No

Were the caregivers and/or investigators blinded from knowledge which intervention each animal received during the experiment?

Was blinding of caregivers and investigators ensured, and was it unlikely that their blinding could have been broken?

Unclear

Were animals selected at random for outcome assessment?

Did the investigators randomly pick an animal during outcome assessment, or did they use a random component in the sequence generation for outcome assessment?

Unclear

Was the outcome assessor blinded?

1. Was blinding of the outcome assessor ensured,
   and was it unlikely that blinding could have been broken?

   Unclear

2. Was the outcome assessor not blinded, but do review authors judge that the outcome is not likely to be influenced by lack of blinding?

   Unclear, but outcome influence will be influenced if blinding is not performed.

Were incomplete outcome data adequately addressed? (*)

1. Were all animals included in the analysis?

   Yes

2. Were the reasons for missing outcome data unlikely to be related to true outcome? (e.g., technical failure)

   No missing data.

3. Are missing outcome data balanced in numbers across intervention groups, with similar reasons for missing data across groups?

No missing data.

3. Are missing outcome data imputed using appropriate methods?

No missing data.

Are reports of the study free of selective outcome reporting? (*)

1. Was the study protocol available and were all of the study's pre-specified primary and secondary outcomes reported in the current manuscript?

Yes

2.. Was the study protocol not available, but was it clear that the published report included all expected outcomes (i.e. comparing methods and results section)?

Not applicable

Was the study apparently free of other problems that could result in high risk of bias?

1. Was the study free of contamination (pooling drugs)?

Unclear

2. Was the study free of inappropriate influence of funders

Yes

3. Was the study free of unit of analysis errors?

Yes

4. Were design-specific risks of bias absent?

Yes

Panahifar et al. (8)

Was the allocation sequence adequately generated and applied?

5. Did the investigators describe a random component in the sequence generation process?

   Yes

Were the groups similar at baseline, or were they adjusted for the confounders in the analysis?

6. Was the distribution of relevant baseline characteristics balanced for the intervention and control groups?

   Unclear

7. If relevant, did the investigators adequately adjust for unequal distribution of some relevant baseline characteristics in the analysis?

   Unclear

8. Was the timing of disease induction adequate?

   Yes

Was the allocation to the different groups adequately concealed during?

Could the investigator allocating the animals to intervention or control group not foresee assignment due to one of the following or equivalent methods?

Unclear

Were the animals randomly housed during the experiment?

3. Did the authors randomly place the cages or animals within the animal room/facility?

   Unclear

4. Is it unlikely that the outcome or the outcome measurement was influenced by not randomly housing the animals?

   No

Were the caregivers and/or investigators blinded from knowledge which intervention each animal received during the experiment?

Was blinding of caregivers and investigators ensured, and was it unlikely that their blinding could have been broken?

Unclear

Were animals selected at random for outcome assessment?

Did the investigators randomly pick an animal during outcome assessment, or did they use a random component in the sequence generation for outcome assessment?

Unclear

Was the outcome assessor blinded?

1. Was blinding of the outcome assessor ensured, and was it unlikely that blinding could have been broken?

   Yes

2. Was the outcome assessor not blinded, but do review authors judge that the outcome is not likely to be influenced by lack of blinding?

   No

Were incomplete outcome data adequately addressed? (*)

1. Were all animals included in the analysis?

   No

2. Were the reasons for missing outcome data unlikely to be related to true outcome? (e.g., technical failure)

   No

3. Are missing outcome data balanced in numbers across intervention groups, with similar reasons for missing data across groups?

   Yes

3. Are missing outcome data imputed using appropriate methods?

   Yes

Are reports of the study free of selective outcome reporting? (*)

1. Was the study protocol available and were all of the study's pre-specified primary and secondary outcomes reported in the current manuscript?

   Yes

2.. Was the study protocol not available, but was it clear that the published report included all expected outcomes (i.e. comparing methods and results section)?

   Not applicable

Was the study apparently free of other problems that could result in high risk of bias?

1. Was the study free of contamination (pooling drugs)?

   Unclear

2. Was the study free of inappropriate influence of funders

   Yes

3. Was the study free of unit of analysis errors?

Yes

4. Were design-specific risks of bias absent?

Yes

Nagy et al. (9)

Was the allocation sequence adequately generated and applied?

1. Did the investigators describe a random component in the sequence generation process?

   Yes

Were the groups similar at baseline, or were they adjusted for the confounders in the analysis?

2. Was the distribution of relevant baseline characteristics balanced for the intervention and control groups?

   Unclear

3. If relevant, did the investigators adequately adjust for unequal distribution of some relevant baseline characteristics in the analysis?

   Not applicable

4. Was the timing of disease induction adequate?

   Yes

Was the allocation to the different groups adequately concealed during?

Could the investigator allocating the animals to intervention or control group not foresee assignment due to one of the following or equivalent methods?

Unclear

Were the animals randomly housed during the experiment?

1. Did the authors randomly place the cages or animals within the animal room/facility?

   Unclear

2. Is it unlikely that the outcome or the outcome measurement was influenced by not randomly housing the animals?

   No

Were the caregivers and/or investigators blinded from knowledge which intervention each animal received during the experiment?

Was blinding of caregivers and investigators ensured, and was it unlikely that their blinding could have been broken?

Unclear

Were animals selected at random for outcome assessment?

Did the investigators randomly pick an animal during outcome assessment, or did they use a random component in the sequence generation for outcome assessment?

Unclear

Was the outcome assessor blinded?

1. Was blinding of the outcome assessor ensured, and was it unlikely that blinding could have been broken?

   Yes

2. Was the outcome assessor not blinded, but do review authors judge that the outcome is not likely to be influenced by lack of blinding?

   No

Were incomplete outcome data adequately addressed? (*)

1. Were all animals included in the analysis?

   No

2. Were the reasons for missing outcome data unlikely to be related to true outcome? (e.g., technical failure)

   No

3. Are missing outcome data balanced in numbers across intervention groups, with similar reasons for missing data across groups?

   Yes

3. Are missing outcome data imputed using appropriate methods?

   Yes

Are reports of the study free of selective outcome reporting? (*)

1. Was the study protocol available and were all of the study's pre-specified primary and secondary outcomes reported in the current manuscript?

   Yes

2.. Was the study protocol not available, but was it clear that the published report included all expected outcomes (i.e. comparing methods and results section)?

   Not applicable

Was the study apparently free of other problems that could result in high risk of bias?

1. Was the study free of contamination (pooling drugs)?

   Yes

2. Was the study free of inappropriate influence of funders

   Yes

3. Was the study free of unit of analysis errors?

   Yes

4. Were design-specific risks of bias absent?

   Yes

Tu et al. (10)

Was the allocation sequence adequately generated and applied?

1. Did the investigators describe a random component in the sequence generation process?

   No

Were the groups similar at baseline, or were they adjusted for the confounders in the analysis?

2. Was the distribution of relevant baseline characteristics balanced for the intervention and control groups?

   Unclear

3. If relevant, did the investigators adequately adjust for unequal distribution of some relevant baseline characteristics in the analysis?

   Unclear

4. Was the timing of disease induction adequate?

   Unclear

Was the allocation to the different groups adequately concealed during?

Could the investigator allocating the animals to intervention or control group not foresee assignment due to one of the following or equivalent methods?

Unclear

Were the animals randomly housed during the experiment?

5. Did the authors randomly place the cages or animals within the animal room/facility?

   Unclear

6. Is it unlikely that the outcome or the outcome measurement was influenced by not randomly housing the animals?

Unclear

Were the caregivers and/or investigators blinded from knowledge which intervention each animal received during the experiment?

Was blinding of caregivers and investigators ensured, and was it unlikely that their blinding could have been broken?

Unclear

Were animals selected at random for outcome assessment?

Did the investigators randomly pick an animal during outcome assessment, or did they use a random component in the sequence generation for outcome assessment?

Unclear

Was the outcome assessor blinded?

1. Was blinding of the outcome assessor ensured, and was it unlikely that blinding could have been broken?

   Unclear

2. Was the outcome assessor not blinded, but do review authors judge that the outcome is not likely to be influenced by lack of blinding?

   Unclear, if blinding in primary outcome assessment was not performed, this will influence outcome.

Were incomplete outcome data adequately addressed? (*)

1. Were all animals included in the analysis?

   Unclear

2. Were the reasons for missing outcome data unlikely to be related to true outcome? (e.g., technical failure)

   Unclear

3. Are missing outcome data balanced in numbers across intervention groups, with similar reasons for missing data across groups?

Unclear

3. Are missing outcome data imputed using appropriate methods?

   Unclear

Are reports of the study free of selective outcome reporting? (*)

1. Was the study protocol available and were all of the study's pre-specified primary and secondary outcomes reported in the current manuscript?

   No

2.. Was the study protocol not available, but was it clear that the published report included all expected outcomes (i.e. comparing methods and results section)?

   Unclear

Was the study apparently free of other problems that could result in high risk of bias?

1. Was the study free of contamination (pooling drugs)?

   Unclear

2. Was the study free of inappropriate influence of funders

   Yes

3. Was the study free of unit of analysis errors?

   Yes

4. Were design-specific risks of bias absent?

   Yes

Wen et al. (11)

Was the allocation sequence adequately generated and applied?

7. Did the investigators describe a random component in the sequence generation process?

   Yes

Were the groups similar at baseline, or were they adjusted for the confounders in the analysis?

8. Was the distribution of relevant baseline characteristics balanced for the intervention and control groups?

   Yes

9. If relevant, did the investigators adequately adjust for unequal distribution of some relevant baseline characteristics in the analysis?

   Not applicable

10. Was the timing of disease induction adequate?

    Yes

Was the allocation to the different groups adequately concealed during?

Could the investigator allocating the animals to intervention or control group not foresee assignment due to one of the following or equivalent methods?

Unclear

Were the animals randomly housed during the experiment?

11. Did the authors randomly place the cages or animals within the animal room/facility?

    Unclear

12. Is it unlikely that the outcome or the outcome measurement was influenced by not randomly housing the animals?

    No

Were the caregivers and/or investigators blinded from knowledge which intervention each animal received during the experiment?

Was blinding of caregivers and investigators ensured, and was it unlikely that their blinding could have been broken?

Unclear

Were animals selected at random for outcome assessment?

Did the investigators randomly pick an animal during outcome assessment, or did they use a random component in the sequence generation for outcome assessment?

Unclear

Was the outcome assessor blinded?

1. Was blinding of the outcome assessor ensured, and was it unlikely that blinding could have been broken?

   Yes

2. Was the outcome assessor not blinded, but do review authors judge that the outcome is not likely to be influenced by lack of blinding?

   No

Were incomplete outcome data adequately addressed? (*)

1. Were all animals included in the analysis?

   Unclear

2. Were the reasons for missing outcome data unlikely to be related to true outcome? (e.g., technical failure)

   Unclear

3. Are missing outcome data balanced in numbers across intervention groups, with similar reasons for missing data across groups?

   Unclear

3. Are missing outcome data imputed using appropriate methods?

   Unclear

Are reports of the study free of selective outcome reporting? (*)

1. Was the study protocol available and were all of the study's pre-specified primary and secondary outcomes reported in the current manuscript?

   Yes

2.. Was the study protocol not available, but was it clear that the published report included all expected outcomes (i.e. comparing methods and results section)?

   Not applicable

Was the study apparently free of other problems that could result in high risk of bias?

1. Was the study free of contamination (pooling drugs)?

   Unclear

2. Was the study free of inappropriate influence of funders

   Yes

3. Was the study free of unit of analysis errors?

   Yes

4. Were design-specific risks of bias absent?

   Yes

Li et al. (12)

Was the allocation sequence adequately generated and applied?

1. Did the investigators describe a random component in the sequence generation process?

   Yes

Were the groups similar at baseline, or were they adjusted for the confounders in the analysis?

1. Was the distribution of relevant baseline characteristics balanced for the intervention and control groups?

   Unclear

2. If relevant, did the investigators adequately adjust for unequal distribution of some relevant baseline characteristics in the analysis?

   Unclear

3. Was the timing of disease induction adequate?

   Yes

Was the allocation to the different groups adequately concealed during?

Could the investigator allocating the animals to intervention or control group not foresee assignment due to one of the following or equivalent methods?

Unclear

Were the animals randomly housed during the experiment?

1. Did the authors randomly place the cages or animals within the animal room/facility?

   Unclear

2. Is it unlikely that the outcome or the outcome measurement was influenced by not randomly housing the animals?

   No

Were the caregivers and/or investigators blinded from knowledge which intervention each animal received during the experiment?

Was blinding of caregivers and investigators ensured, and was it unlikely that their blinding could have been broken?

Unclear

Were animals selected at random for outcome assessment?

Did the investigators randomly pick an animal during outcome assessment, or did they use a random component in the sequence generation for outcome assessment?

Unclear

Was the outcome assessor blinded?

1. Was blinding of the outcome assessor ensured, and was it unlikely that blinding could have been broken?

   Unclear

2. Was the outcome assessor not blinded, but do review authors judge that the outcome is not likely to be influenced by lack of blinding?

   Unclear, but outcome influence will be influenced if blinding is not performed.

Were incomplete outcome data adequately addressed? (*)

1. Were all animals included in the analysis?

   Yes

2. Were the reasons for missing outcome data unlikely to be related to true outcome? (e.g., technical failure)

   No missing data.

3. Are missing outcome data balanced in numbers across intervention groups, with similar reasons for missing data across groups?

   No missing data.

3. Are missing outcome data imputed using appropriate methods?

   No missing data.

Are reports of the study free of selective outcome reporting? (*)

1. Was the study protocol available and were all of the study's pre-specified primary and secondary outcomes reported in the current manuscript?

   Yes

2.. Was the study protocol not available, but was it clear that the published report included all expected outcomes (i.e. comparing methods and results section)?

   Not applicable

Was the study apparently free of other problems that could result in high risk of bias?

1. Was the study free of contamination (pooling drugs)?

   Unclear

2. Was the study free of inappropriate influence of funders

   Yes

3. Was the study free of unit of analysis errors?

   Yes

4. Were design-specific risks of bias absent?

   Yes

Liu et al. (13)

Was the allocation sequence adequately generated and applied?

1. Did the investigators describe a random component in the sequence generation process?

   Yes

Were the groups similar at baseline, or were they adjusted for the confounders in the analysis?

1. Was the distribution of relevant baseline characteristics balanced for the intervention and control groups?

   Unclear

2. If relevant, did the investigators adequately adjust for unequal distribution of some relevant baseline characteristics in the analysis?

   Unclear

3. Was the timing of disease induction adequate?

   Yes

Was the allocation to the different groups adequately concealed during?

Could the investigator allocating the animals to intervention or control group not foresee assignment due to one of the following or equivalent methods?

Unclear

Were the animals randomly housed during the experiment?

1. Did the authors randomly place the cages or animals within the animal room/facility?

   Unclear

2. Is it unlikely that the outcome or the outcome measurement was influenced by not randomly housing the animals?

   No

Were the caregivers and/or investigators blinded from knowledge which intervention each animal received during the experiment?

Was blinding of caregivers and investigators ensured, and was it unlikely that their blinding could have been broken?

Unclear

Were animals selected at random for outcome assessment?

Did the investigators randomly pick an animal during outcome assessment, or did they use a random component in the sequence generation for outcome assessment?

Unclear

Was the outcome assessor blinded?

1. Was blinding of the outcome assessor ensured, and was it unlikely that blinding could have been broken?

   Unclear

2. Was the outcome assessor not blinded, but do review authors judge that the outcome is not likely to be influenced by lack of blinding?

   Unclear, but outcome influence will be influenced if blinding is not performed.

Were incomplete outcome data adequately addressed? (*)

1. Were all animals included in the analysis?

   Unclear

2. Were the reasons for missing outcome data unlikely to be related to true outcome? (e.g., technical failure)

   Unclear

3. Are missing outcome data balanced in numbers across intervention groups, with similar reasons for missing data across groups?

   Unclear

3. Are missing outcome data imputed using appropriate methods?

   Unclear

Are reports of the study free of selective outcome reporting? (*)

1. Was the study protocol available and were all of the study's pre-specified primary and secondary outcomes reported in the current manuscript?

   Yes

2.. Was the study protocol not available, but was it clear that the published report included all expected outcomes (i.e. comparing methods and results section)?

   Not applicable

Was the study apparently free of other problems that could result in high risk of bias?

1. Was the study free of contamination (pooling drugs)?

   Unclear

2. Was the study free of inappropriate influence of funders

   Yes

3. Was the study free of unit of analysis errors?

   Yes

4. Were design-specific risks of bias absent?

   Yes

Dai et al. (14)

Was the allocation sequence adequately generated and applied?

1. Did the investigators describe a random component in the sequence generation process?

Yes

Were the groups similar at baseline, or were they adjusted for the confounders in the analysis?

1. <u>Was the distribution of relevant baseline characteristics balanced for the intervention and control groups?</u>

   Unclear

2. <u>If relevant, did the investigators adequately adjust for unequal distribution of some relevant baseline characteristics in the analysis?</u>

   Unclear

3. <u>Was the timing of disease induction adequate?</u>

   Yes

Was the allocation to the different groups adequately concealed during?

<u>Could the investigator allocating the animals to intervention or control group not foresee assignment due to one of the following or equivalent methods?</u>

Unclear

Were the animals randomly housed during the experiment?

1. <u>Did the authors randomly place the cages or animals within the animal room/facility?</u>

   Unclear

2. <u>Is it unlikely that the outcome or the outcome measurement was influenced by not randomly housing the animals?</u>

   No

Were the caregivers and/or investigators blinded from knowledge which intervention each animal received during the experiment?

Was blinding of caregivers and investigators ensured, and was it unlikely that their blinding could have been broken?

Unclear

Were animals selected at random for outcome assessment?

Did the investigators randomly pick an animal during outcome assessment, or did they use a random component in the sequence generation for outcome assessment?

Unclear

Was the outcome assessor blinded?

1. Was blinding of the outcome assessor ensured, and was it unlikely that blinding could have been broken?

   Yes

2. Was the outcome assessor not blinded, but do review authors judge that the outcome is not likely to be influenced by lack of blinding?

   No

Were incomplete outcome data adequately addressed? (*)

1. Were all animals included in the analysis?

   Yes

2. Were the reasons for missing outcome data unlikely to be related to true outcome? (e.g., technical failure)

   No missing data.

3. Are missing outcome data balanced in numbers across intervention groups, with similar reasons for missing data across groups?

   No missing data.

3. Are missing outcome data imputed using appropriate methods?

   No missing data.

Are reports of the study free of selective outcome reporting? (*)

1. Was the study protocol available and were all of the study's pre-specified primary and secondary outcomes reported in the current manuscript?

   Yes

2.. Was the study protocol not available, but was it clear that the published report included all expected outcomes (i.e. comparing methods and results section)?

   Not applicable

Was the study apparently free of other problems that could result in high risk of bias?

1. Was the study free of contamination (pooling drugs)?

   No, but unlikely that the drugs used will influence the results

2. Was the study free of inappropriate influence of funders

   Yes

3. Was the study free of unit of analysis errors?

   Yes

4. Were design-specific risks of bias absent?

   Yes

Jean et al. (15)

Was the allocation sequence adequately generated and applied?

1. Did the investigators describe a random component in the sequence generation process?

   No

Were the groups similar at baseline, or were they adjusted for the confounders in the analysis?

2. Was the distribution of relevant baseline characteristics balanced for the intervention and control groups?

   Yes

3. If relevant, did the investigators adequately adjust for unequal distribution of some relevant baseline characteristics in the analysis?

   Not applicable

4. Was the timing of disease induction adequate?

   Yes

Was the allocation to the different groups adequately concealed during?

Could the investigator allocating the animals to intervention or control group not foresee assignment due to one of the following or equivalent methods?

Unclear

Were the animals randomly housed during the experiment?

1. Did the authors randomly place the cages or animals within the animal room/facility?

   Unclear

2. Is it unlikely that the outcome or the outcome measurement was influenced by not randomly housing the animals?

No

Were the caregivers and/or investigators blinded from knowledge which intervention each animal received during the experiment?

Was blinding of caregivers and investigators ensured, and was it unlikely that their blinding could have been broken?

Unclear

Were animals selected at random for outcome assessment?

Did the investigators randomly pick an animal during outcome assessment, or did they use a random component in the sequence generation for outcome assessment?

Unclear

Was the outcome assessor blinded?

1. Was blinding of the outcome assessor ensured, and was it unlikely that blinding could have been broken?

   Yes

2. Was the outcome assessor not blinded, but do review authors judge that the outcome is not likely to be influenced by lack of blinding?

   No

Were incomplete outcome data adequately addressed? (*)

1. Were all animals included in the analysis?

   Unclear

2. Were the reasons for missing outcome data unlikely to be related to true outcome? (e.g., technical failure)

   Unclear

3. Are missing outcome data balanced in numbers across intervention groups, with similar reasons for missing data across groups?

Unclear

3. Are missing outcome data imputed using appropriate methods?

   Unclear

Are reports of the study free of selective outcome reporting? (*)

1. Was the study protocol available and were all of the study's pre-specified primary and secondary outcomes reported in the current manuscript?

   Yes

2.. Was the study protocol not available, but was it clear that the published report included all expected outcomes (i.e. comparing methods and results section)?

   Not applicable

Was the study apparently free of other problems that could result in high risk of bias?

1. Was the study free of contamination (pooling drugs)?

   No, but risk of biasing of the drugs used is low

2. Was the study free of inappropriate influence of funders

   Yes

3. Was the study free of unit of analysis errors?

   Yes

4. Were design-specific risks of bias absent?

   Yes

Jiang et al. (16)

Was the allocation sequence adequately generated and applied?

1. Did the investigators describe a random component in the sequence generation process?

   No

Were the groups similar at baseline, or were they adjusted for the confounders in the analysis?

2. Was the distribution of relevant baseline characteristics balanced for the intervention and control groups?

   Unclear

3. If relevant, did the investigators adequately adjust for unequal distribution of some relevant baseline characteristics in the analysis?

   Unclear

4. Was the timing of disease induction adequate?

   Yes

Was the allocation to the different groups adequately concealed during?

Could the investigator allocating the animals to intervention or control group not foresee assignment due to one of the following or equivalent methods?

Unclear

Were the animals randomly housed during the experiment?

3. Did the authors randomly place the cages or animals within the animal room/facility?

   Unclear

4. Is it unlikely that the outcome or the outcome measurement was influenced by not randomly housing the animals?

No

Were the caregivers and/or investigators blinded from knowledge which intervention each animal received during the experiment?

Was blinding of caregivers and investigators ensured, and was it unlikely that their blinding could have been broken?

Unclear

Were animals selected at random for outcome assessment?

Did the investigators randomly pick an animal during outcome assessment, or did they use a random component in the sequence generation for outcome assessment?

Unclear

Was the outcome assessor blinded?

1. Was blinding of the outcome assessor ensured, and was it unlikely that blinding could have been broken?

   Yes

2. Was the outcome assessor not blinded, but do review authors judge that the outcome is not likely to be influenced by lack of blinding?

   No

Were incomplete outcome data adequately addressed? (*)

1. Were all animals included in the analysis?

   No

2. Were the reasons for missing outcome data unlikely to be related to true outcome? (e.g., technical failure)

   No

3. Are missing outcome data balanced in numbers across intervention groups, with similar reasons for missing data across groups?

Yes

3. Are missing outcome data imputed using appropriate methods?

   Yes

Are reports of the study free of selective outcome reporting? (*)

1. Was the study protocol available and were all of the study's pre-specified primary and secondary outcomes reported in the current manuscript?

   Yes

2.. Was the study protocol not available, but was it clear that the published report included all expected outcomes (i.e. comparing methods and results section)?

   Not applicable

Was the study apparently free of other problems that could result in high risk of bias?

1. Was the study free of contamination (pooling drugs)?

   Unclear

2. Was the study free of inappropriate influence of funders

   Yes

3. Was the study free of unit of analysis errors?

   Yes

4. Were design-specific risks of bias absent?

   Yes

Dong et al. (17)

Was the allocation sequence adequately generated and applied?

1. Did the investigators describe a random component in the sequence generation process?

   No

Were the groups similar at baseline, or were they adjusted for the confounders in the analysis?

2. Was the distribution of relevant baseline characteristics balanced for the intervention and control groups?

   Yes

3. If relevant, did the investigators adequately adjust for unequal distribution of some relevant baseline characteristics in the analysis?

   Not applicable

4. Was the timing of disease induction adequate?

   Yes

Was the allocation to the different groups adequately concealed during?

Could the investigator allocating the animals to intervention or control group not foresee assignment due to one of the following or equivalent methods?

Unclear

Were the animals randomly housed during the experiment?

1. Did the authors randomly place the cages or animals within the animal room/facility?

   Unclear

2. Is it unlikely that the outcome or the outcome measurement was influenced by not randomly housing the animals?

No

Were the caregivers and/or investigators blinded from knowledge which intervention each animal received during the experiment?

Was blinding of caregivers and investigators ensured, and was it unlikely that their blinding could have been broken?

Unclear

Were animals selected at random for outcome assessment?

Did the investigators randomly pick an animal during outcome assessment, or did they use a random component in the sequence generation for outcome assessment?

Unclear

Was the outcome assessor blinded?

1. Was blinding of the outcome assessor ensured, and was it unlikely that blinding could have been broken?

   Yes

2. Was the outcome assessor not blinded, but do review authors judge that the outcome is not likely to be influenced by lack of blinding?

   No

Were incomplete outcome data adequately addressed? (*)

1. Were all animals included in the analysis?

   Unclear

2. Were the reasons for missing outcome data unlikely to be related to true outcome? (e.g., technical failure)

   Unclear

3. Are missing outcome data balanced in numbers across intervention groups, with similar reasons for missing data across groups?

Unclear

3. Are missing outcome data imputed using appropriate methods?

   Unclear

Are reports of the study free of selective outcome reporting? (*)

1. Was the study protocol available and were all of the study's pre-specified primary and secondary outcomes reported in the current manuscript?

   Yes

2.. Was the study protocol not available, but was it clear that the published report included all expected outcomes (i.e. comparing methods and results section)?

   Not applicable

Was the study apparently free of other problems that could result in high risk of bias?

1. Was the study free of contamination (pooling drugs)?

   No, but risk of biasing of the drugs used is low

2. Was the study free of inappropriate influence of funders

   Yes

3. Was the study free of unit of analysis errors?

   Yes

4. Were design-specific risks of bias absent?

   Yes

Wen et al. (18)

Was the allocation sequence adequately generated and applied?

1. Did the investigators describe a random component in the sequence generation process?

   Yes

Were the groups similar at baseline, or were they adjusted for the confounders in the analysis?

2. Was the distribution of relevant baseline characteristics balanced for the intervention and control groups?

   Yes

3. If relevant, did the investigators adequately adjust for unequal distribution of some relevant baseline characteristics in the analysis?

   Not applicable

4. Was the timing of disease induction adequate?

   Yes

Was the allocation to the different groups adequately concealed during?

Could the investigator allocating the animals to intervention or control group not foresee assignment due to one of the following or equivalent methods?

Unclear

Were the animals randomly housed during the experiment?

5. Did the authors randomly place the cages or animals within the animal room/facility?

   Unclear

6. Is it unlikely that the outcome or the outcome measurement was influenced by not randomly housing the animals?

No

Were the caregivers and/or investigators blinded from knowledge which intervention each animal received during the experiment?

Was blinding of caregivers and investigators ensured, and was it unlikely that their blinding could have been broken?

Unclear

Were animals selected at random for outcome assessment?

Did the investigators randomly pick an animal during outcome assessment, or did they use a random component in the sequence generation for outcome assessment?

Unclear

Was the outcome assessor blinded?

1. Was blinding of the outcome assessor ensured, and was it unlikely that blinding could have been broken?

   Unclear

2. Was the outcome assessor not blinded, but do review authors judge that the outcome is not likely to be influenced by lack of blinding?

   Unclear

Were incomplete outcome data adequately addressed? (*)

1. Were all animals included in the analysis?

   No

2. Were the reasons for missing outcome data unlikely to be related to true outcome? (e.g., technical failure)

   No

3. Are missing outcome data balanced in numbers across intervention groups, with similar reasons for missing data across groups?

Unclear

3. <u>Are missing outcome data imputed using appropriate methods?</u>

   Unclear

Are reports of the study free of selective outcome reporting? (*)

1. <u>Was the study protocol available and were all of the study's pre-specified primary and secondary outcomes reported in the current manuscript?</u>

   Yes

2.. <u>Was the study protocol not available, but was it clear that the published report included all expected outcomes (i.e. comparing methods and results section)?</u>

   Not applicable

Was the study apparently free of other problems that could result in high risk of bias?

1. <u>Was the study free of contamination (pooling drugs)?</u>

   No, but risk of biasing of the drugs lis low

2. <u>Was the study free of inappropriate influence of funders</u>

   Yes

3. <u>Was the study free of unit of analysis errors?</u>

   Yes

4. <u>Were design-specific risks of bias absent?</u>

   Yes

Janssen et al. (19)

Was the allocation sequence adequately generated and applied?

1. Did the investigators describe a random component in the sequence generation process?

   Yes

Were the groups similar at baseline, or were they adjusted for the confounders in the analysis?

2. Was the distribution of relevant baseline characteristics balanced for the intervention and control groups?

   Yes

3. If relevant, did the investigators adequately adjust for unequal distribution of some relevant baseline characteristics in the analysis?

   Not applicable

4. Was the timing of disease induction adequate?

   Yes

Was the allocation to the different groups adequately concealed during?

Could the investigator allocating the animals to intervention or control group not foresee assignment?

Unclear

Were the animals randomly housed during the experiment?

5. Did the authors randomly place the cages or animals within the animal room/facility?

   Unclear

6. Is it unlikely that the outcome or the outcome measurement was influenced by not randomly housing the animals?

No

Were the caregivers and/or investigators blinded from knowledge which intervention each animal received during the experiment?

Was blinding of caregivers and investigators ensured, and was it unlikely that their blinding could have been broken?

Unclear

Were animals selected at random for outcome assessment?

Did the investigators randomly pick an animal during outcome assessment, or did they use a random component in the sequence generation for outcome assessment?

Unclear

Was the outcome assessor blinded?

1. Was blinding of the outcome assessor ensured, and was it unlikely that blinding could have been broken?

   Yes

2. Was the outcome assessor not blinded, but do review authors judge that the outcome is not likely to be influenced by lack of blinding?

   No

Were incomplete outcome data adequately addressed? (*)

1. Were all animals included in the analysis?

   No

2. Were the reasons for missing outcome data unlikely to be related to true outcome? (e.g., technical failure)

   No

3. Are missing outcome data balanced in numbers across intervention groups, with similar reasons for missing data across groups?

Yes

3. Are missing outcome data imputed using appropriate methods?

   Yes

Are reports of the study free of selective outcome reporting? (*)

1. Was the study protocol available and were all of the study's pre-specified primary and secondary outcomes reported in the current manuscript?

   Yes

2.. Was the study protocol not available, but was it clear that the published report included all expected outcomes (i.e. comparing methods and results section)?

   Not applicable

Was the study apparently free of other problems that could result in high risk of bias?

1. Was the study free of contamination (pooling drugs)?

   No but low risk of bias of the drugs used

2. Was the study free of inappropriate influence of funders

   Yes

3. Was the study free of unit of analysis errors?

   Yes

4. Were design-specific risks of bias absent?

   Yes

Tellegen et al. (20)

Was the allocation sequence adequately generated and applied?

1. Did the investigators describe a random component in the sequence generation process?

   Yes

Were the groups similar at baseline, or were they adjusted for the confounders in the analysis?

7. Was the distribution of relevant baseline characteristics balanced for the intervention and control groups?

   Yes

8. If relevant, did the investigators adequately adjust for unequal distribution of some relevant baseline characteristics in the analysis?

   Not applicable

9. Was the timing of disease induction adequate?

   Yes

Was the allocation to the different groups adequately concealed during?

Could the investigator allocating the animals to intervention or control group not foresee assignment due to one of the following or equivalent methods?

Unclear

Were the animals randomly housed during the experiment?

10. Did the authors randomly place the cages or animals within the animal room/facility?

    Unclear

11. Is it unlikely that the outcome or the outcome measurement was influenced by not randomly housing the animals?

No

Were the caregivers and/or investigators blinded from knowledge which intervention each animal received during the experiment?

Was blinding of caregivers and investigators ensured, and was it unlikely that their blinding could have been broken?

Unclear

Were animals selected at random for outcome assessment?

Did the investigators randomly pick an animal during outcome assessment, or did they use a random component in the sequence generation for outcome assessment?

Unclear

Was the outcome assessor blinded?

1. Was blinding of the outcome assessor ensured, and was it unlikely that blinding could have been broken?

   Yes

2. Was the outcome assessor not blinded, but do review authors judge that the outcome is not likely to be influenced by lack of blinding?

   No

Were incomplete outcome data adequately addressed? (*)

1. Were all animals included in the analysis?

   Unclear

2. Were the reasons for missing outcome data unlikely to be related to true outcome? (e.g., technical failure)

   Unclear

3. Are missing outcome data balanced in numbers across intervention groups, with similar reasons for missing data across groups?

Unclear

3. Are missing outcome data imputed using appropriate methods?

   Unclear

Are reports of the study free of selective outcome reporting? (*)

1. Was the study protocol available and were all of the study's pre-specified primary and secondary outcomes reported in the current manuscript?

   Yes

2.. Was the study protocol not available, but was it clear that the published report included all expected outcomes (i.e. comparing methods and results section)?

   Not applicable

Was the study apparently free of other problems that could result in high risk of bias?

1. Was the study free of contamination (pooling drugs)?

   No, but the bias risk of drugs used is low

2. Was the study free of inappropriate influence of funders

   Yes

3. Was the study free of unit of analysis errors?

   Yes

4. Were design-specific risks of bias absent?

   Yes

Liu et al. (21)

Was the allocation sequence adequately generated and applied?

1. Did the investigators describe a random component in the sequence generation process?

   Yes

Were the groups similar at baseline, or were they adjusted for the confounders in the analysis?

2. Was the distribution of relevant baseline characteristics balanced for the intervention and control groups?

   Unclear

3. If relevant, did the investigators adequately adjust for unequal distribution of some relevant baseline characteristics in the analysis?

   Unclear

4. Was the timing of disease induction adequate?

   Yes

Was the allocation to the different groups adequately concealed during?

Could the investigator allocating the animals to intervention or control group not foresee assignment due to one of the following or equivalent methods?

Unclear

Were the animals randomly housed during the experiment?

5. Did the authors randomly place the cages or animals within the animal room/facility?

   Unclear

6. Is it unlikely that the outcome or the outcome measurement was influenced by not randomly housing the animals?

   No

Were the caregivers and/or investigators blinded from knowledge which intervention each animal received during the experiment?

Was blinding of caregivers and investigators ensured, and was it unlikely that their blinding could have been broken?

Unclear

Were animals selected at random for outcome assessment?

Did the investigators randomly pick an animal during outcome assessment, or did they use a random component in the sequence generation for outcome assessment?

Unclear

Was the outcome assessor blinded?

1. Was blinding of the outcome assessor ensured, and was it unlikely that blinding could have been broken?

   Yes

2. Was the outcome assessor not blinded, but do review authors judge that the outcome is not likely to be influenced by lack of blinding?

   No

Were incomplete outcome data adequately addressed? (*)

1. Were all animals included in the analysis?

   Yes

2. Were the reasons for missing outcome data unlikely to be related to true outcome? (e.g., technical failure)

   No missing data

3. Are missing outcome data balanced in numbers across intervention groups, with similar reasons for missing data across groups?

   No missing data

3. Are missing outcome data imputed using appropriate methods?

No missing data

Are reports of the study free of selective outcome reporting? (*)

1. Was the study protocol available and were all of the study's pre-specified primary and secondary outcomes reported in the current manuscript?

   Yes

2.. Was the study protocol not available, but was it clear that the published report included all expected outcomes (i.e. comparing methods and results section)?

   Not applicable

Was the study apparently free of other problems that could result in high risk of bias?

1. Was the study free of contamination (pooling drugs)?

   Unclear

2. Was the study free of inappropriate influence of funders

   Yes

3. Was the study free of unit of analysis errors?

   Yes

4. Were design-specific risks of bias absent?

   Yes

1. Mastbergen SC, Marijnissen AC, Vianen ME, Zoer B, van Roermund PM, Bijlsma JW, et al. Inhibition of COX-2 by celecoxib in the canine groove model of osteoarthritis. Rheumatology (Oxford). 2006;45(4):405-13.

2. Huh JE, Baek YH, Kim YJ, Lee JD, Choi DY, Park DS. Protective effects of butanol fraction from Betula platyphyla var. japonica on cartilage alterations in a rabbit collagenase-induced osteoarthritis. J Ethnopharmacol. 2009;123(3):515-21.
3. Jones MD, Tran CW, Li G, Maksymowych WP, Zernicke RF, Doschak MR. In vivo microfocal computed tomography and micro-magnetic resonance imaging evaluation of antiresorptive and antiinflammatory drugs as preventive treatments of osteoarthritis in the rat. Arthritis Rheum. 2010;62(9):2726-35.
4. Fukai A, Kamekura S, Chikazu D, Nakagawa T, Hirata M, Saito T, et al. Lack of a chondroprotective effect of cyclooxygenase 2 inhibition in a surgically induced model of osteoarthritis in mice. Arthritis Rheum. 2012;64(1):198-203.
5. Ou Y, Tan C, An H, Jiang D, Quan Z, Tang K, et al. Selective COX-2 inhibitor ameliorates osteoarthritis by repressing apoptosis of chondrocyte. Med Sci Monit. 2012;18(6):BR247-52.
6. Ashkavand Z, Malekinejad H, Amniattalab A, Rezaei-Golmisheh A, Vishwanath BS. Silymarin potentiates the anti-inflammatory effects of Celecoxib on chemically induced osteoarthritis in rats. Phytomedicine. 2012;19(13):1200-5.
7. Moon SJ, Park JS, Jeong JH, Yang EJ, Park MK, Kim EK, et al. Augmented chondroprotective effect of coadministration of celecoxib and rebamipide in the monosodium iodoacetate rat model of osteoarthritis. Arch Pharm Res. 2013;36(1):116-24.
8. Panahifar A, Jaremko JL, Tessier AG, Lambert RG, Maksymowych WP, Fallone BG, et al. Development and reliability of a multi-modality scoring system for evaluation of disease progression in pre-clinical models of osteoarthritis: celecoxib may possess disease-modifying properties. Osteoarthritis Cartilage. 2014;22(10):1639-50.
9. Nagy E, Vajda E, Vari C, Sipka S, Farr AM, Horvath E. Meloxicam ameliorates the cartilage and subchondral bone deterioration in monoiodoacetate-induced rat osteoarthritis. PeerJ. 2017;5:e3185.
10. Tu M, Yang M, Yu N, Zhen G, Wan M, Liu W, et al. Inhibition of cyclooxygenase-2 activity in subchondral bone modifies a subtype of osteoarthritis. Bone Res. 2019;7:29.
11. Wen ZH, Lin YY, Chang YC, Tang CC, Hsieh SP, Lee HP, et al. The COX-2 inhibitor etoricoxib reduces experimental osteoarthritis and nociception in rats: The roles of TGF-beta1 and NGF expressions in chondrocytes. Eur J Pain. 2020;24(1):209-22.
12. Li Z, Meng D, Li G, Xu J, Tian K, Li Y. Celecoxib Combined with Diacerein Effectively Alleviates Osteoarthritis in Rats via Regulating JNK and p38MAPK Signaling Pathways. Inflammation. 2015;38(4):1563-72.
13. Liu B, Ji C, Shao Y, Liang T, He J, Jiang H, et al. Etoricoxib decreases subchondral bone mass and attenuates biomechanical properties at the early stage of osteoarthritis in a mouse model. Biomed Pharmacother. 2020;127:110144.
14. Dai MW, Chu JG, Tian FM, Song HP, Wang Y, Zhang YZ, et al. Parathyroid hormone(1-34) exhibits more comprehensive effects than celecoxib in cartilage metabolism and maintaining subchondral bone micro-architecture in meniscectomized guinea pigs. Osteoarthritis Cartilage. 2016;24(6):1103-12.
15. Jean YH, Wen ZH, Chang YC, Hsieh SP, Tang CC, Wang YH, et al. Intra-articular injection of the cyclooxygenase-2 inhibitor parecoxib attenuates osteoarthritis progression in anterior cruciate ligament-transected knee in rats: role of excitatory amino acids. Osteoarthritis Cartilage. 2007;15(6):638-45.
16. Jiang D, Zou J, Huang L, Shi Q, Zhu X, Wang G, et al. Efficacy of intra-articular injection of celecoxib in a rabbit model of osteoarthritis. Int J Mol Sci. 2010;11(10):4106-13.

17. Dong J, Jiang D, Wang Z, Wu G, Miao L, Huang L. Intra-articular delivery of liposomal celecoxib-hyaluronate combination for the treatment of osteoarthritis in rabbit model. Int J Pharm. 2013;441(1-2):285-90.
18. Wen ZH, Tang CC, Chang YC, Huang SY, Chen CH, Wu SC, et al. Intra-articular injection of the selective cyclooxygenase-2 inhibitor meloxicam (Mobic) reduces experimental osteoarthritis and nociception in rats. Osteoarthritis Cartilage. 2013;21(12):1976-86.
19. Janssen M, Timur UT, Woike N, Welting TJ, Draaisma G, Gijbels M, et al. Celecoxib-loaded PEA microspheres as an auto regulatory drug-delivery system after intra-articular injection. J Control Release. 2016;244(Pt A):30-40.
20. Tellegen AR, Rudnik-Jansen I, Pouran B, de Visser HM, Weinans HH, Thomas RE, et al. Controlled release of celecoxib inhibits inflammation, bone cysts and osteophyte formation in a preclinical model of osteoarthritis. Drug Deliv. 2018;25(1):1438-47.
21. Liu P, Gu L, Ren L, Chen J, Li T, Wang X, et al. Intra-articular injection of etoricoxib-loaded PLGA-PEG-PLGA triblock copolymeric nanoparticles attenuates osteoarthritis progression. Am J Transl Res. 2019;11(11):6775-89.
