# Supplementary material for: Chondroprotective Actions of Selective COX-2 Inhibitors In Vivo: A Systematic Review
Source: Int J Mol Sci. 2020 Sep 22;21(18):6962. doi: 10.3390/ijms21186962 (PMC7555215; doi:10.3390/ijms21186962)
Supplement: Supplementary file 1 [file ijms-21-06962-s001.zip › ijms-931722-supplymentary revised done/biasassesmentclinicalstudies.pdf]

Supplementary Data: Cochrane risk of bias tool for clinical studies

Tindall et al. (1)

	**Authors' judgement**	**Support for judgment**
Random sequence generation (selection bias)	High risk	No randomization was performed (open-label study)
Allocation concealment (selection bias)	High risk	No adequate concealment of allocations (open-label study)
Blinding of participants and personnel (Performance Bias)	High risk	No blinding (open-label study)
Blinding of Outcome Assesement (Detection Bias)	High risk	Participants received the same treatment, no blinding in radiographic outcome assessment between two dosages celecoxib
Incomplete outcome data (Attrition Bias)	Unclear risk	Exact numbers of missing data not reported
Selective Reporting (Reporting Bias)	Low risk	For all groups, all radiographic outcome measuruments are given
Other bias	None	None

De Boer et al. (2)

	**Authors' judgement**	**Support for judgment**
Random sequence generation (selection bias)	High risk	No randomization was performed (open-label study)
Allocation concealment (selection bias)	High risk	No adequate concealment of allocations (open-label study)
Blinding of participants and personnel (Performance Bias)	High risk	No blinding (open-label study)
Blinding of Outcome Assesment (Detection Bias)	Intermediate risk	Blinding was performed to the source of cartilage for histological analysis. For biochemical analysis no blinding was perforemd
Incomplete outcome data (Attrition Bias)	Low risk	No missing data
Selective Reporting (Reporting Bias)	Low risk	Results of the original variables measured are given
Other bias	None	None

Raynauld et al. (3)

	**Authors' judgement**	**Support for judgment**
Random sequence generation (selection bias)	High risk	No randomization was performed (open-label study)
Allocation concealment (selection bias)	High risk	No adequate concealment of allocations (open-label study)
Blinding of participants and personnel (Performance Bias)	High risk	No blinding (open-label study)
Blinding of Outcome Assesment (Detection Bias)	Low risk	Blinding was performed when analyzing primary outcome (MRI variables)
Incomplete outcome data (Attrition Bias)	Unclear risk	Missing data not reported
Selective Reporting (Reporting Bias)	Low risk	Results of the original variables measured are given
Other bias	None	None

Sawitzke et al. (4)

1. Tindall EA, Sharp JT, Burr A, Katz TK, Wallemark CB, Verburg K, et al. A 12-month, multicenter, prospective, open-label trial of radiographic analysis of disease progression in osteoarthritis of the knee or hip in patients receiving celecoxib. Clin Ther. 2002;24(12):2051-63.

2. de Boer TN, Huisman AM, Polak AA, Niehoff AG, van Rinsum AC, Saris D, et al. The chondroprotective effect of selective COX-2 inhibition in osteoarthritis: ex vivo evaluation of

	**Authors' judgement**	**Support for judgment**
Random sequence generation (selection bias)	Unclear risk	Randomization method not specified.
Allocation concealment (selection bias)	Unclear risk	The method used to conceal the allocation sequence is not described
Blinding of participants and personnel (Performance Bias)	Unclear risk	Blinding for treatment is described, but no description is given how participant blinding was performed
Blinding of Outcome Assesment (Detection Bias)	Low risk	Blinding was performed in primary outcome assessment
Incomplete outcome data (Attrition Bias)	Low risk	Missing data are reported and no differences between randomized groups in missing data
Selective Reporting (Reporting Bias)	Low risk	Results of the original variables measured are given
Other bias	None	None

human cartilage tissue after in vivo treatment. Osteoarthritis Cartilage. 2009;17(4):482-8.
3. Raynauld JP, Martel-Pelletier J, Beaulieu A, Bessette L, Morin F, Choquette D, et al. An open-label pilot study evaluating by magnetic resonance imaging the potential for a disease-modifying effect of celecoxib compared to a modelized historical control cohort in the treatment of knee osteoarthritis. Semin Arthritis Rheum. 2010;40(3):185-92.
4. Sawitzke AD, Shi H, Finco MF, Dunlop DD, Bingham CO, 3rd, Harris CL, et al. The effect of glucosamine and/or chondroitin sulfate on the progression of knee osteoarthritis: a report from the glucosamine/chondroitin arthritis intervention trial. Arthritis Rheum. 2008;58(10):3183-91.
